# Cervico-Occipital Neuralgia

**Published:** 1873-11-01

**Authors:** A. W. Lueck

**Affiliations:** Mayville, Wisconsin


### CERVICO-OCCIPITAL NEURALGIA.

PAPER READ BEFORE THE ROCK RIVER MEDICAL SOCIETY, BY A. W. LUECK, M.D., OF MAYVILLE, WISCONSIN.

On the morning of August 7th, 1873, we were called to Mrs. H——, aged 50 years, who was stated to be in great distress. On arrival, we found our patient evidently in great suffering. She was tossing herself about in the bed, and was sometimes screaming aloud with pain.

We learned, that some days previous, while working in the field and being in a state of perspiration, she was chilled, towards evening, by a shower. Since that time she felt a pain in the nape of her neck, extending around it towards the right side. This pain increased little by little, until last night it had been almost unbearable, being somewhat of a paroxysmal character. Manipulation of the muscles of the neck did not increase the pain, hence we could exclude *torticollis* in our diagnosis, and place the case among the neuralgias. However, our patient had had articular rheumatism, so we concluded to put her first on anti-rheumatic treatment. But, to relieve her present suffering, we injected in the areola tissue of the neck about one-fourth grain sulph. morph., and one forty-eighth grain sulph. atropia. The other medicine being iodide and bromide of potassium, vin. colch. rad., magnes. sulph., and pulv. Doveri.

August 8th, at 7 P. M.—We found our patient in a condition worse than yesterday. The hypodermic injection had relieved the pain for about five hours; after that time the paroxysms came on with increased vigor. We now made another injection in the arm, and ordered four leeches to the effected part, followed by warm fomentation. Internally, she was ordered to take one of the following pills every three hours.

℞.—Quin. sulph. grs. xl.
Iodiform, grs. xx.
Ferri redacti, grs. xv.
Veratria, gr. j.

Make into 20 pills.

She should also take tinct. opii deodorat gtts. x., as often as might be necessary to relieve the pain.

August 9th, 3 P. M.—On opening the door, our patient greeted us with a smile. She was sitting comfortable in the bed, saying that she had not suffered any pain since we last saw her. By some misunderstanding, she had, since 3 o'clock this morning, taken eight pills, instead of four, as we directed, and three doses of the opium.

We directed to take no more pills until the next day, and then only four during the day.

The most interesting question now is, which of these medicines cured our patient? The hypodermic injection of morphia and atrophine certainly relieved her; but the first injection showed, that this relief was only temporarily. The local bleeding may have some claim in the cure; but since only two of the leeches had drawn, we think this cannot amount to much. The same we must say of the quinine and opium. We must, therefore, in this case, ascribe the curative effect to iodiform and veratria; which of these two is the most prominent, we are not prepared to say. However, iodiform, having served us so well in other forms of neuralgia, we would again recommend it to the attention of the members of this Society.

